# On the Role of Adenosine A2A Receptor Gene Transcriptional Regulation in Parkinson’s Disease

**DOI:** 10.3389/fnins.2019.00683

**Published:** 2019-07-10

**Authors:** Anastasia Falconi, Alessandra Bonito-Oliva, Martina Di Bartolomeo, Marcella Massimini, Francesco Fattapposta, Nicoletta Locuratolo, Enrico Dainese, Esterina Pascale, Gilberto Fisone, Claudio D’Addario

**Affiliations:** ^1^Faculty of Bioscience, University of Teramo, Teramo, Italy; ^2^Department of Neuroscience, Karolinska Institute, Stockholm, Sweden; ^3^Department of Human Neurosciences, Sapienza University, Rome, Italy; ^4^Department of Medical-Surgical Sciences and Biotechnologies, Sapienza University, Rome, Italy; ^5^Department of Clinical Neuroscience, Karolinska Institute, Stockholm, Sweden

**Keywords:** Parkinson’s disease, adenosine A2A receptor, 6-hydroxydopamine, peripheral blood mononuclear cells, DNA methylation, histone modifications

## Abstract

Adenosine A2A receptors (A2ARs) have attracted considerable attention as an important molecular target for the design of Parkinson’s disease (PD) therapeutic compounds. Here, we studied the transcriptional regulation of the A2AR gene in human peripheral blood mononuclear cells (PBMCs) obtained from PD patients and in the striatum of the well-validated, 6-hydroxydopamine (6-OHDA)-induced PD mouse model. We report an increase in A2AR mRNA expression and protein levels in both human cells and mice striata, and in the latter we could also observe a consistent reduction in DNA methylation at gene promoter and an increase in histone H3 acetylation at lysine 9. Of particular relevance in clinical samples, we also observed higher levels in the receptor gene expression in younger subjects, as well as in those with less years from disease onset, and less severe disease according to clinical scores. In conclusion, the present findings provide further evidence of the relevant role of A2AR in PD and, based on the clinical data, highlight its potential role as disease biomarker for PD especially at the initial stages of disease development. Furthermore, our preclinical results also suggest selective epigenetic mechanisms targeting gene promoter as tool for the development of new treatments.

## Introduction

Parkinson’s disease (PD), the second most common neurodegenerative disorder after Alzheimer’s disease, affects approximately 1% of the population over 60 (Ozansoy and Basak, 2013). The pathological hallmark of PD is the degeneration of nigrostriatal dopaminergic neurons and the consequent loss of dopaminergic input to the basal ganglia, which gives rise to well-defined motor symptoms, including bradykinesia, rigidity, muscular stiffness, tremor, poor posture and balance, and sensory motor integration deficits (Marsden, 2000; Obeso et al., 2000). Epidemiological studies reveal that less than 10% of PD cases are familial, while most are sporadic. The etiology of the disease remains poorly understood and is likely the result of an intricate interplay between genetic, epigenetic, and environmental factors, among others. At present, there are no FDA-approved disease-modifying treatments.

Nowadays, dopamine replacement treatments represent the best therapy available to alleviate PD symptoms. The dopamine precursor L-3,4-dihydroxyphenylalanine (L-DOPA) is the most efficacious and commonly prescribed anti-parkinsonian drug. However, its prolonged use is limited by the occurrence of a number of debilitating side effects (LeWitt, 2015).

Among the different possible targets for symptomatic treatments, the adenosine A2ARs have attracted considerable interest. A2ARs are enriched in the medium spiny neurons (MSN) of the striatum, which is the main component of the basal ganglia (Jarvis and Williams, 1989; Svenningsson et al., 1997; Rosin et al., 1998). Importantly, A2ARs are selectively expressed on the MSNs of the indirect striatopallidal pathway (Schiffmann et al., 1991; Fink et al., 1992), where they antagonize dopamine D2 receptor-mediated transmission (Schiffmann et al., 2007).

In line with these findings, several preclinical and clinical studies point to A2ARs antagonists as a promising non-dopaminergic therapy for PD (Feigin, 2003; Pinna et al., 2005; Schwarzschild et al., 2006). Moreover, oral administration of the A2ARs antagonist KW-6002 showed a significant neuroprotective effect in a rat model of PD characterized by dopamine depletion achieved by administration of the toxin 6-hydroxydopamine (6-OHDA) (Ikeda et al., 2002).

Adenosine A2A receptors gene expression was found to be up-regulated in the striata of rats with a 6-OHDA lesion (Pinna et al., 2002), and in the putamen and peripheral blood mononuclear cells (PBMCs) of PD and mild cognitive impairment patients (Calon et al., 2004; Varani et al., 2010; Casetta et al., 2014). These observations were confirmed by PET studies, showing enhanced striatal A2ARs levels in PD patients (Ramlackhansingh et al., 2011). However, others have reported a reduction of A2ARs in the anterior and posterior caudate nucleus and anterior dorsal putamen of individuals with PD (Hurley et al., 2000), or no changes in the striata of rats with a 6-OHDA lesion (Kaelin-Lang et al., 2000; Tomiyama et al., 2004).

It has been suggested that DNA methylation, an epigenetic mark associated with gene repression (Jones, 2012), might have a key role in regulating A2AR gene transcription (Buira et al., 2010a, b). In line with this hypothesis, reduced DNA methylation in the 5′UTR region of A2AR gene was observed in advanced PD cases (Villar-Menéndez et al., 2014). Another well-studied mechanism of epigenetic regulation is the post-translational modification of histone tails. Increased histone acetylation has also been observed in experimental models of PD as well as in the brain of PD patients (Park et al., 2016), leading to the hypothesis that drugs that affect histone acetylation would have therapeutic effects (Song et al., 2011; Harrison and Dexter, 2013). So far, there are no studies that selectively focus on the role of histone modifications on A2AR gene transcription regulation in PD.

Based on this background, the present study deeply investigates the A2AR gene transcriptional regulation via epigenetic mechanisms in PD. To this aim, we employed a multidisciplinary approach, based on the use of clinical (PBMCs of PD patients) and preclinical samples (brain tissue of 6-OHDA-lesioned mice).

## Experimental Procedures

### Subjects

For this study we enrolled 73 outpatients attending the Neurological Clinic in La Sapienza University, Rome, on stable pharmacological treatment. Diagnosis of sporadic PD was based on clinical symptoms according to the United Kingdom. Brain Bank Criteria for PD (Hughes et al., 1992). Patients showing a comorbid substance or alcohol abuse in the previous 2 months were ruled out. Exclusion criteria included signs of atypical parkinsonism, diagnosis of mental retardation or dementia (Mini-Mental State Examination score <23.8). The variables collected included: smoking habit, age at onset of PD, clinical form (Tremor-dominant TD, Non-Tremor-dominant NTD), motor disability by means of the Unified Parkinson’s Disease rating Scale-subset III (UPDRS III), disease stage according to Hoehn & Yahr scale, duration of disease, levodopa equivalent daily dose (LEDD) calculated according to Tomlinson (Tomlinson et al., 2010). We also selected sex and age-matched healthy subjects as a control group. The exclusion criteria were: alcohol and substance abuse, neurological disorders, family history of movement disorders. Subjects suffering from metabolic disorders, severe hypertension or systemic autoimmune diseases were also excluded. No statistical difference between patients and control groups emerged in either hypertension or dyslipidemia cases. The study was approved by the local ethics committee. Written informed consent was obtained from all study participants. Demographic and clinical characteristics for the study samples are shown in Table 1.

**TABLE 1:** Demographic and clinical characteristics of PD patients and controls.

**Characteristic**	**Controls *n* = 32**	**PD *n* = 73**
Male (%)	16 (50%)	40 (55%)
Smokers (%)	11 (34%)	23 (31%)
Age (yrs, mean ± SD)	71.5 ± 6.8	68.8 ± 7.2
Age at Onset (yrs, mean ± SD)	NA	62.3 ± 7.7
Disease duration (yrs, mean ± SD)	NA	7.6 ± 11.6
LEDD at last visit (mg/die, mean ± SD)	NA	509.5 ± 35
UPDRS III score	NA	14.1 ± 5.8
H&Y scale (mean ± SD)	NA	1.78 ± 0.5
1		21
1.5		3
2		36
2.5		12
3		1
Tremor dominant (%)	NA	30 (41%)

*H&Y, Hohen &Yahr scale; UPDRS III, unified Parkinson’s disease rating scale-subset III; LEDD, levodopa equivalent daily dose.*

### Animals

Male C57BL/6J mice (25–30 g; Taconic, Tornbjerg, Denmark) were housed under a 12 h light/dark cycle with food and water *ad libitum*. Experiments were carried out in accordance with the guidelines of Research Ethics Committee of Karolinska Institutet, Swedish Animal Welfare Agency and European Communities Council Directive 86/609/EEC.

### 6-OHDA Lesion and Brain Dissection

Mice were anesthetized with a mixture of Hypnorm^®^ (VetaPharma Ltd., Leed, United Kingdom), midazolam (5 mg/ml) (Hameln Pharmaceuticals GmbH, Hameln, Germany), and water (1:1:2 in a volume of 10 ml/kg) and mounted in a stereotaxic frame (David Kopf Instruments, Tujunga, CA, United States). 6-OHDA was dissolved in 0.02% ascorbic acid in saline at the concentration of 3.0 g of freebase 6-OHDA/l. Each mouse received two unilateral injections of vehicle (Sham, unlesioned) or 6-OHDA (2 μl/injection) into the right dorsal striatum as previously described (Santini et al., 2007), according to the following coordinates (in mm) (Franklin and Paxinos, 2008): anterior-posterior +1, medial-lateral −2.1, dorsal-ventral −3.2 and anterior-posterior +0.3, medial-lateral −2.3, and dorsal-ventral −3.2. Three weeks after surgery, the mice were killed by decapitation, their heads were cooled in liquid nitrogen for 6 s and striata were dissected out on an ice-cold surface and snap frozen in liquid nitrogen. The success of the lesion was assessed at the end of the experiments by measuring striatal levels of tyrosine hydroxylase (TH) in Sham *vs* unlesioned mice by Western Blot (see below). The success of the lesion was defined by ≥80% TH decrease and only the mice that met these criteria were included in the analysis.

### Molecular Biology Studies

**FIGURE 1:**
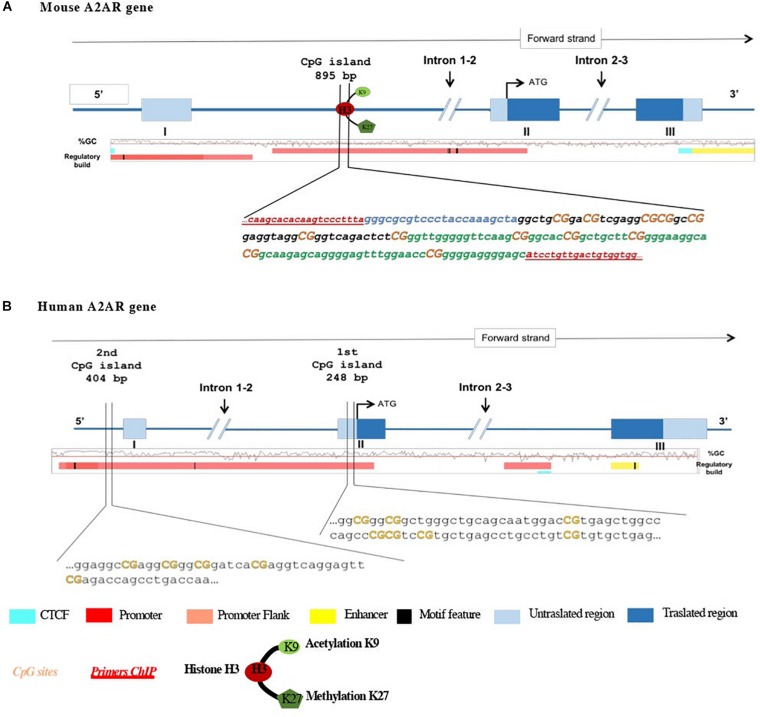
Schematic representation of **(A)** mouse and **(B)** human A2AR gene promoters and the 5′ upstream region. Translation start code (ATG), exons and introns, CpG island, and histone modifications studied are depicted. In mouse A2AR gene promoter, two regions (region 1 in black and region 2 in green) localized in the same CpG island. Orange bold text indicates the CpG sites analyzed.

To evaluate regulation of A2ARs transcription, we analyzed mRNA and protein levels, as well as epigenetic modifications at A2AR gene promoter such as DNA methylation and histone modifications (Figure 1).

### Real-Time Quantitative PCR (RT-qPCR)

Peripheral blood mononuclear cells were isolated from the peripheral blood of control subjects and PD patients by Fycoll-Paque PLUS density gradient medium according to manufacturer’s instructions (GE Healthcare, Bio-Sciences AB Uppsala-Sweden). Total RNA was extracted from PBMCs and single striatum samples (Chomczynski and Sacchi, 2006) and checked for integrity by electrophoresis. RNA concentrations were then measured by spectrophotometry and just samples reporting an OD 260:280 ratio >2 were subjected to DNAse treatment and converted to cDNA with a commercially available kit (Thermo Fisher Scientific, Waltham, MA, United States). Diluted cDNAs were thus used to assess A2AR mRNA relative abundance by RT-qPCR, using SensiFast No-Rox Kit (Bioline) using the DNA Engine Opticon-2 detection system (Biorad, CA, United States). β-actin and GAPDH genes, properly validated to confirm that in our experimental conditions their expression was not affected, were used as reference genes to normalize the data.

Sequences of the primers used for PCR amplification are listed in Table 2. In a final volume of 15 μl, we used 2 μl of cDNA, 7,5 μl of SensiFAST SYBR, and 10 pmol of each primer. Duplicate samples were run and PCR conditions were: 95°C for 10 s, 60°C for 30 s, and 72 for 30 s. A2ARs relative expression was calculated by Delta-Delta Ct (ΔΔCT) method and converted to 2^-ΔΔ*Ct*^ for statistical analysis (Livak and Schmittgen, 2001).

**TABLE 2:** Imers employed during the quantitative polymerase chain reaction, chromatin immunoprecipitation, and DNA methylation assays.

		**Gene expression**	
**Species**	**Gene**	**Forward**	**Reverse**
Human	β-actin	GCACCAGATCATGTTTGAGACCT	CCATACACGATGCCAGTGGT
	GAPDH	CAGCCTCAAGATCATCAGCA	TGTGGTCATGAGTCGTTCCA
	A2AR	CATCCCGCTCCGGTACAATG	TGGTTCTTGCCCTCCTTTGG
Mouse	β-actin	AACGGGAAGCTCACTGGCAT	GTCTCAAACATGATCTGGGTC
	GAPDH	AACGGGAAGCTCACTGGCAT	AACGGGAAGCTCACTGGCAT
	A2AR	AGAGCAAGAGGCAGGTATCTC	CCCAAAGGCTTTCTCACGGA
	A2AR ChIP	CAAGCACACAAGTCCCTTTA	CAAGCACACAAGTCCCTTTA

		**DNA methylation**	
		**Region 1**	**Region 2**
Human A2AR	Forward	TTTGGGTAGGGTTGGGAGTT	AGGTGGAGGTTGTAGTGA
	Reverse	CCCAACACACCAACACATT	CCACACTCCCTCTTTTCTTT
	Sequencing	GGTAGGGTTGGGAGTTA	GGAGGTTGTAGTGAG
Mouse A2AR	Forward	PM00220542 Qiagen, Hilden, Germany	GGAGGGGATTGAATTTGTAAGTATA
	Reverse		CCAAACACCCACCCTATTATC
	Sequencing		GAGGTAGGAGGGTTAGAT

### DNA Methylation Analysis by Pyrosequencing

Genomic DNA, obtained from human PBMCs and striatum tissues, was bisulfite-treated according to manufacturer’s instructions (Zymo Research, Irvine, CA, United States). Methylation status of human and mouse A2AR GENE was assessed using pyrosequencing of the bisulfite-converted DNA as previously reported (Cifani et al., 2015; Pucci et al., 2015).

Pyrosequencing primers were designed to focus on a series of CpG dinucleotides part of the CpG island located in two different regions both in clinical samples and in mice brain tissues (see Figure 1 and Table 2 for details). Bisulfite-treated DNA was amplified by PyroMark PCR Kit (Qiagen, Germany) under these PCR conditions: 95°C for 15 min; 45 cycles of 94°C for 30 s; 56°C for 30 s; 72°C for 30 s; and final step of 72°C for 10 min. Following PCR products verification by agarose electrophoresis, pyrosequencing methylation analysis was conducted using the PyroMark Q24 Software (Qiagen, Germany), which allows for each CpG site quantitative comparisons of the methylation percentage.

### Chromatin Immunoprecipitation (ChIP)

Dahl and Collas protocol, with minor modifications, was used to prepare chromatin from mice frozen tissues as previously described (Dahl and Collas, 2007). Briefly, to cross-link proteins to DNA, formaldehyde was added at a final concentration of 1% in phosphate buffer saline containing a broad-range protease inhibitor cocktail (PIC) (Sigma, St. Louis, MO, United States) and sodium butyrate (Sigma, St. Louis, MO, United States), for 10 min at room temperature. Glycine, used to quench the reaction, was added to a final concentration of 0.125 M and incubating for 5 min at room temperature. Following washing, the samples were lysed using 120 μl of a lysis buffer (50 mM Tris–HCl, pH 8, 10 mM EDTA, 1% SDS) containing PIC and sodium butyrate (20 mM). The samples were incubated on ice and sonicated for 30 s for 6 times, with 30 s pause intervals each sonicated. The lysates were centrifuged at 12,000 *g* for 10 min at 4°C and the supernatants transferred into a chilled tube, leaving around 30 μl of buffer with the pellet. Lysis buffer (30 μl) was added. DNA fragments ranging in size from 200 to 500 bp were analyzed by agarose gel electrophoresis. 20 μl aliquot was used as “input” DNA, for each immunoprecipitation. Chromatin was diluted in 90 μl of RIPA buffer (10 mM Tris–HCl, pH 7.5, 1 mM EDTA, 0.5 mM EGTA, 1% Triton X-100, 0.1% SDS, 0.1% Na-deoxycholate, 140 mM NaCl) plus PIC and incubated overnight by rotation with either antibody previously coated with Protein A beads (Invitrogen, Carlsbad, CA, United States), Histone 3 acetylation at Lysine 9 (H3K9Ac) (PA5 17868, Thermo Fisher Scientific, Carlsbad, CA, United States), or Histone 3 trimethylation at Lysine 27 (H3K27me3) (PA5 17173, Thermo Fisher Scientific, Carlsbad, CA, United States). The beads and associated immune complexes were washed three times with RIPA buffer and once with Tris–EDTA buffer. The immune complexes were eluted with elution buffer (20 mM Tris–HCl, 5 mM EDTA, 50 mM NaCl) containing proteinase K (50 μg/ml) (Qiagen, Valencia, CA, United States) at 68°C for 2 h, and DNA was recovered by NucleoSpin TriPrep (Macherey-Nagel, Germany). Thereafter, to quantify A2AR gene sequences associated with the immunoprecipitated proteins, RT-qPCR was carried out using primers designed with Primer 3 software (Rozen and Skaletsky, 2000; see Table 1). All ChIP data were normalized to the input DNA amounts (Ct values of immunoprecipitated samples were normalized to Ct values obtained from “input”). In addition, results on DNA from lesioned animals were normalized by the DNA data obtained from control animals (control group).

### Western Blot

Total cellular lysates from human PBMCs and mice tissues were prepared with different procedures. PBMCs were lysed in RIPA buffer whereas 0.3 gr of striatum tissue sample of lesioned and control animals were homogenized in T-PER lysis buffer (PIERCE, Rockford, IL, United States) containing 1% NP-40 detergent solution, 5% glycerol, 1 mM EDTA and 0.1% PIC (Sigma-Aldrich, Milan, Italy). Both human and mouse samples were sonicated and then centrifuged at 5000 *g* for 30 min at 4°C. Protein concentrations were measured according to Bradford method (1976). For each sample, 50 μg of human proteins and 30 μg of mouse proteins were electrophoresed (12% acrylamide gels) and transferred to PVDF membranes (Amersham Biosciences, Piscataway, NJ, United States). Membranes, blocked with a solution of 5% non-fat dry milk for 20 min and with 5% BSA for 40 min at room temperature, were incubated with a rabbit anti-A2A polyclonal antibody (PA1-042, Thermo Fisher Scientific, Carlsbad, CA, United States, 1:5000 in blocking solution) and a rabbit anti-GAPDH monoclonal antibody (2118S, Cell Signaling, Danvers, MA, United States, 1:5000 in blocking solution) overnight in cold room. GAPDH was used to normalize samples. Antibody against TH (Chemicon, Temecula, CA, 1:1000) was used in mice samples to assess the severity of the 6-OHDA lesions. Finally, the membranes were incubated with specific horseradish peroxidase-conjugated secondary anti-rabbit antibody for 1 h at room temperature (AP307P, Millipore, Darmstadt, Germany, 1:10000 in blocking solution). The antigen-antibody complex was detected by enhanced chemiluminescence (ECL, Amersham Biosciences) and the intensities of the immunoreactive bands were quantified by densitometric analysis using the ImageJ software (NIH, Bethesda, MD, United States).

### Statistical Analysis

Non-parametric statistic (Mann-Whitney *U* test) was used to compare lesioned vs. unlesioned striata, as well as the human PD and control samples. For correlation analysis, Spearman’s coefficient was used. *p* < 0.05 was considered statistically significant. All the mentioned tests were performed using GraphPad Prism version 6.00 (GraphPad Software, San Diego, CA, United States).

## Results

### Human Subjects

**FIGURE 2:**
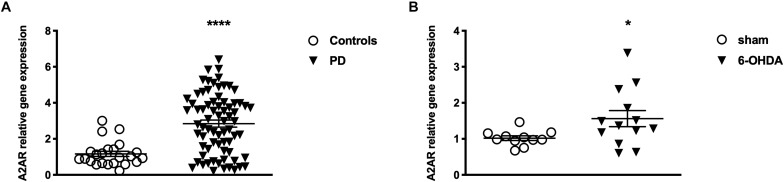
Levels of A2AR mRNA **(A)** in PBMC from controls (*n* = 22) and PD patients (*n* = 25) and **(B)** in the striatum of Sham (*n* = 11) and 6-OHDA (*n* = 13) animals. Bars represents 2^-ΔΔ*Ct*^ value calculated by Delta-Delta Ct (ΔΔCt) method. ^*^p < 0.05 and ^****^p < 0.0001 vs. respective control groups.

**FIGURE 3:**
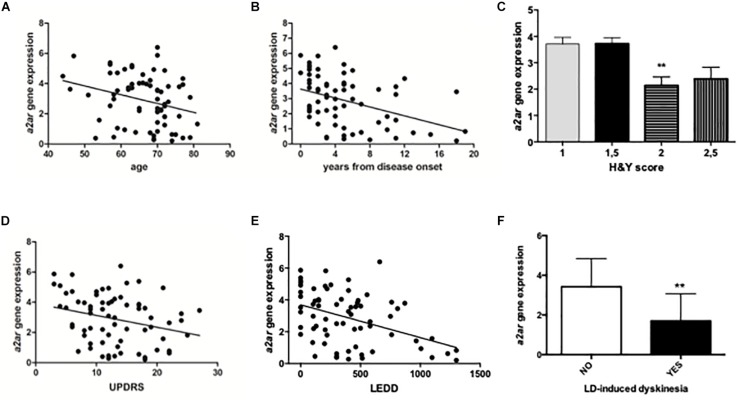
Data stratification and correlation analysis between A2AR mRNA levels in PD human subjects and age **(A**, years from disease onset **(B)**, H&Y score **(C)**, UPDRS score **(D)**, LEDD **(E)**, and LD-induced dyskinesia **(F)**. ^∗∗^*p* < 0.01 vs. respective control groups.

Patients and controls were age and gender matched to allow consistent comparisons. RT-PCR analysis revealed significantly higher A2AR mRNA levels in PD patients when compared to healthy controls (PD: 2.84 ± 0.14; Controls: 1.13 ± 0.12 *p* < 0.0001 Mann Whitney test) (Figure 2A). Moreover, data stratification analysis showed a significant correlation between A2AR gene expression and age of the subjects (Spearman *r* = −0.2931; *p* = 0.014), years from disease onset (Spearman *r* = −0.4046; *p* = 0.001), as well as Hoehn & Yahr (H&Y) (*p* < 0.01 score 2 vs. score 1), UPDRS (Spearman *r* = −0.2752; *p* = 0.0211) scores, and LEDD (Spearman *r* = −0.3902; *p* = 0.001) (Figure 3). On the other hand, no correlation was observed between A2AR gene expression and age in controls (Spearman *r* = 0.2399; *p* = 0.3226). Correlation analysis survived Dunn’s multiple comparisons test for age, UPDRS score, and LEDD. Finally, in a multiple linear regression analysis A2AR mRNA levels were found to be related to age (*p* = 0.0343) and gender (*p* = 0.0335), as well as LEDD (*p* = 0.0005).

**FIGURE 4:**
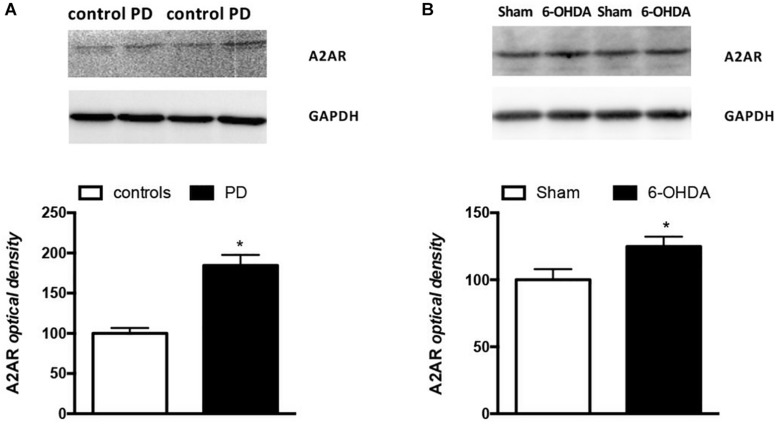
Analysis of A2AR protein levels in PBMCs from **(A)** PD patients (*n* = 5) and controls (*n* = 4) and **(B)** Sham (*n* = 12) and 6-OHDA (*n* = 10) mice striata. Representative immunoblots of PBMCs and striata lysates reacted with specific anti-A2AR or anti-GAPDH antibodies are shown above the bars for both mice and humans. Values, expressed as means ± standard error of the mean (SEM), were normalized by GAPDH and taking the control and sham groups as 100, respectively for **(A,B)**. ^*^p < 0.05 vs. respective control groups.

It is interesting to note that also A2AR density, expressed as A2AR/GAPDH ratio, was significantly higher in PD patients when compared to controls (Controls: 100% ± 6.67; PD: 184.5% ± 13.07; *p =* 0.0159 Mann Whitney test) (Figure 4A). DNA methylation analyzed at A2AR promoter in two different CpG islands did not show any difference between PD and controls (Table 3).

**TABLE 3:** DNA methylation levels (mean ± SEM) at seven cytosine-guanine dinucleotide (CpG) sites (Region 1) and at five cytosine-guanine dinucleotide (CpG) sites (Region 2), within the human A2AR gene promoter, in PBMCs of control (*n* = 22), and PD subjects (*n* = 25).

**Region 1**	**CpG site 1**	**CpG site 2**	**CpG site 3**	**CpG site 4**	**CpG site 5**	**CpG site 6**	**CpG site 7**
CT	97.50 ± 0.07	95.89 ± 0.10	97.97 ± 0.16	100	81.81 ± 0.34	92.62 ± 0.21	93.07 ± 0.37
PD	97.45 ± 0.05	95.83 ± 0.10	97.31 ± 0.27	100	81.32 ± 0.20	93.03 ± 0.23	92.40 ± 0.31
**Region 2**	**CpG site 1**	**CpG site 2**	**CpG site 3**	**CpG site 4**	**CpG site 5**		
CT	92.02 ± 1.03	94.77 ± 0.63	85.81 ± 0.47	75.81 ± 0.47	36.49 ± 4.63		
PD	92.39 ± 0.76	95.51 ± 0.48	85.97 ± 0.75	74.93 ± 0.57	33.27 ± 0.98		

*ANOVAs and subsequent *post hoc* tests did not indicate any significant difference in the level of methylation at CpG sites at the two regions analyzed between PD and control patients.*

### 6-OHDA Mice Model

**FIGURE 5:**
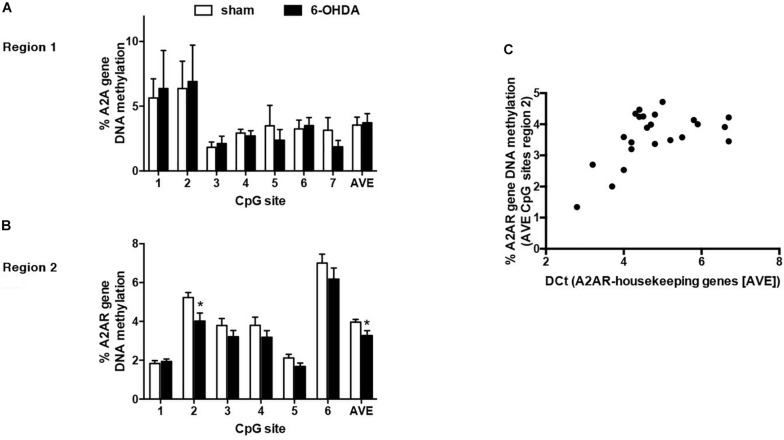
Percentage of DNA methylation assessed with bisulfite pyrosequencing in sham (*n* = 12) and 6-OHDA (*n* = 14) mice striata at A2AR gene promoter: **(A)** Region 1 (7 CpG sites quantified). **(B)** Region 2 (6 CpG sites quantified). Values are expressed as means ± SEM. ^*^p < 0.05 vs. Sham group. **(C)** Correlation between average DNA methylation at 6 CpG sites located in Region 2 and A2AR mRNA levels in mice striata. Data were compared with non-parametric Spearman correlation analysis. ^*^P = 0.0357.

**FIGURE 6:**
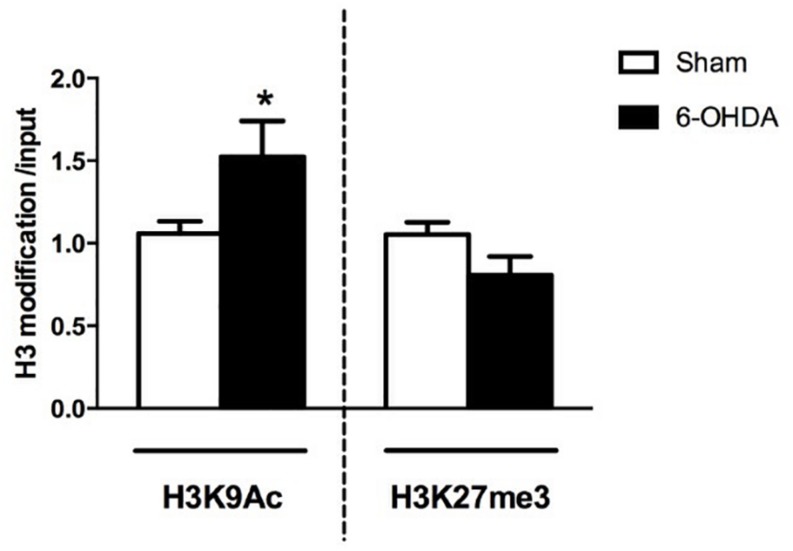
RT-qPCR analysis of H3K9Ac and H3K27me3 immunoprecipitated fragments at A2AR gene promoter. Bars show specific histone modification levels, normalized to total input DNA in Sham (*n* = 11), and 6-OHDA (*n* = 9) animals. Values are expressed as means ± SEM. ^*^p < 0.05 vs. Sham group.

The first result we observed in mice is the reduced TH immunoreactivity to 10.3 ± 1.22% of control Sham-lesioned animals (100.0 ± 3.39%) (see Supplementary Figure S1). Successively, the study of the A2AR transcriptional regulation revealed a significant up-regulation of A2AR mRNA in the striata of 6-OHDA lesioned mice, when compared to control (Sham) mice (Sham: 1.02 ± 0.06; 6-OHDA: 1.56 ± 0.22; *p* = 0.0041 Mann Whitney test) (Figure 2B). In agreement with these findings, we observed a parallel increase of A2AR protein levels in 6-OHDA-lesioned animals when compared to controls (Sham: 100.0 ± 7.89; 6-OHDA: 124.84 ± 7.39; *p =* 0.0425 Mann Whitney test) (Figure 4B). See Supplementary Figure S2 for further details about the analysis of protein levels. We also observed a consistent reduction in DNA methylation at A2AR gene promoter selectively in one of the two regions under study, and specifically in the second CpG site (Sham: 5.06 ± 0.41; 6-OHDA: 4.45 ± 0.60; *p* = 0.025) as well as in the average of the 6 CpG sites analyzed (Sham: 3.97 ± 0.22; 6-OHDA: 3.52 ± 0.35; *p* = 0.031) (Figure 5B). Notably, gene expression and DNA methylation levels were inversely correlated in all samples (Spearman *r* = −0.427, *p* = 0.037) (Figure 5C). No changes were observed in region 1 (Figure 5A). Finally, we report also a significant enrichment of H3K9Ac (a histone mark exerting permissive action on gene transcription) at A2AR GENE promoter, at the level of the same region studied for DNA methylation in 6-OHDA mice (Sham: 1.06 ± 0.07; 6-OHDA: 1.52 ± 0.21; *p* = 0.038) (Figure 6). We also analyzed the levels of the repressive marker, H3K27me3, but we did not observe any significant change (Sham: 1.05 ± 0.07; 6-OHDA: 0.80 ± 0.11; *p* = 0.075 (Figure 6).

## Discussion

This study shows that dopamine depletion, a characteristic trait of PD experimentally induced *in vivo* by 6-OHDA, evokes in mice striata A2AR gene up-regulation as well as increase in protein receptor levels. The same alterations have also been observed in PBMCs from PD patients when compared to healthy controls.

Our data corroborate previous studies showing increased A2AR transcription in the striatum of dopamine-denervated rats (Pinna et al., 2002) and in the putamen of PD patients (Varani et al., 2010) suggesting a similar regulation in the 6-OHDA mouse model of PD as well as in PBMCs from PD subjects. In addition, we provide evidence that these changes are paralleled by a significant increase in A2AR levels, confirming the same change already observed in both 6-OHDA rat model (Bhattacharjee et al., 2011) and clinical samples (Calon et al., 2004; Varani et al., 2010; Ramlackhansingh et al., 2011; Casetta et al., 2014).

Notably, these clinical studies established a correlation between L-DOPA-induced motor complications (i.e., dyskinesia) and increased levels of A2AR. Thus, A2AR density is significantly higher in lymphocytes and neutrophils of dyskinetic than non-dyskinetic patients. Interestingly, L-DOPA-induced dyskinesia is influenced by the degree of dopamine depletion, suggesting that the increase in A2AR expression may be particularly prominent in advanced PD (Varani et al., 2010). Our findings in patients instead show different pattern of changes. In fact, data stratification based on age, as well as years from disease onset, showed higher levels in receptor gene expression in younger patients and in subjects affected for less than a few years, as well as in those with less severe disease even if it should be considered the limited range of H&Y and UPDRS scores. This is in agreement with the results from Villar-Menéndez et al. (2014) showing that the increased A2AR occurs as an early event in PD.

Importantly, these effects in mice are accompanied by consistent changes in two relevant epigenetic marks: a significant reduction in DNA methylation and a significant increase in H3K9Ac at gene promoter. DNA methylation role in A2AR gene regulation was previously reported (Buira et al., 2010a, b; Villar-Menéndez et al., 2014) and we here show in mice striata a significant and selective reduction in DNA methylation clearly correlated with the increase in gene expression.

Moreover, we observed a hitherto undiscovered increase in H3K9Ac, a permissive epigenetic mark, in line with the upregulation of gene expression. Global histone hyperacetylation represents a key epigenetic change in dopaminergic neurons and has been proposed to participate in PD pathogenesis (Villar-Menéndez et al., 2013; Kleiveland, 2015) however this is the first study showing a specific change in H3 acetylation at A2AR gene in the dopamine-depleted striatum. This finding suggests that the reduction in DNA methylation of this gene might be mediated by a local state of acetylation, as previously proposed as a global effect (Cervoni and Szyf, 2001). No changes in DNA methylation levels have been observed in PBMCs from PD subjects when compared to controls. Instead, others reported a reduction of DNA methylation in two CpG sites at A2AR gene promoter in human brain samples (Villar-Menéndez et al., 2014).

In this study we used whole blood samples composed of different cell types with different DNA methylation profiles (Kleiveland, 2015). Therefore, it will be necessary to extend these findings using novel methodological approaches, such as cell-sorting, to fully elucidate the underlying epigenetic regulation of gene expression. However, it is important to underline that PBMCs share with neurons several cellular components and contain the complete epigenetic machinery present in neurons as well as in many other tissues (Joseph et al., 2018; Sen et al., 2018; Zhu et al., 2018). For this reason, their gene expression profile has been recently proposed as a substitute for cerebral markers which, on the other hand, wouldn’t provide enough insight into biochemical detail in order to originate novel and more effective therapeutic intervention (Woelk et al., 2011; Arosio et al., 2014). Moreover, it would also be relevant to evaluate the molecular outcomes in mice PBMCs and compare them to the data obtained from mice brain samples as well as human PBMCs.

In conclusion, our results in mice indicate that loss of dopaminergic innervation to the striatum results in the upregulation of A2AR GENE expression paralleled by selective epigenetic mechanisms, thereby providing new insights into the role of this receptor in PD. Several clinical trials have shown that A2AR antagonists ameliorate the dyskinesia induced by chronic L-DOPA treatment in PD patients (Pinna et al., 2005; Xu et al., 2005; Mizuno and Kondo, 2013) and it is possible that receptor silencing might be an alternative therapy to reduce receptors activity.

Therefore, these data may offer new vistas for therapeutic interventions in PD by targeting histone acetylation and/or DNA methylation selectively at this gene sequence. These data also suggest a possible role of A2AR transcriptional regulation as a biomarker in PD on the basis of the relevant changes occurring at early stages of disease development observed in patient samples.

## Data Availability

All datasets generated for this study are included in the manuscript and/or the Supplementary Files.

## Ethics Statement

Animal Experiments were carried out in accordance with the guidelines of Research Ethics Committee of Karolinska Institutet, Swedish Animal Welfare Agency, and European Communities Council Directive 86/609/EEC. Humans study was approved by the local ethics committee, patient, and control subjects were asked to give their written informed consent to undergo study procedures.

## Author Contributions

CD’A, EP, and GF conceived and designed the experiments. AF, MD, MM, AB-O, NL, and FF conducted the experiments. CD’A, EP, and ED analyzed the data. ED, CD’A, and GF contributed to the reagents, materials, and analysis tools. CD’A, GF, and AF wrote the manuscript.

## Conflict of Interest Statement

The authors declare that the research was conducted in the absence of any commercial or financial relationships that could be construed as a potential conflict of interest.
