# Supplementary material for: On the Role of Adenosine A2A Receptor Gene Transcriptional Regulation in Parkinson’s Disease
Source: Front Neurosci. 2019 Jul 10;13:683. doi: 10.3389/fnins.2019.00683 (PMC6635589; doi:10.3389/fnins.2019.00683)
Supplement: Supplementary file 1 [file Data_Sheet_1.docx]

**Figure S1**

**Legend to Supplementary Figure 1.** Analysis of TH protein levels in sham and 6-OHDA mice striata. Representative immunoblots of striata lysates reacted with specific anti-TH antibody are shown above the bars. Values, expressed as means ± standard error of the mean (SEM) taking the sham group as 100. * p < 0.05 versus respective control groups.


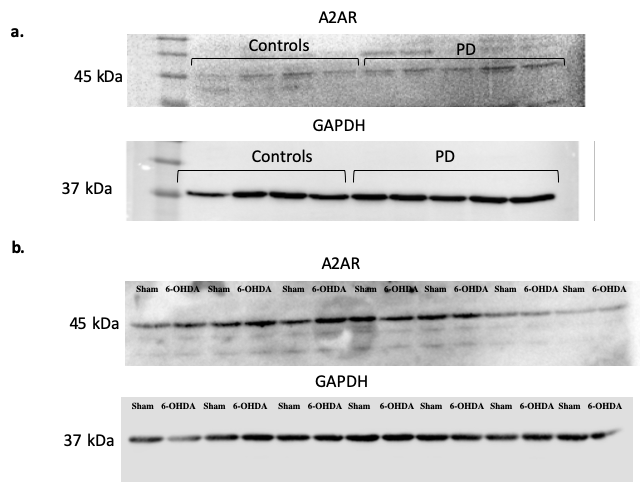


**Figure S2**

**Legend to Supplementary Figure 2.**

Representative immunoblots of PBMCs **(a)** and striata **(b)** lysates reacted with specific anti-A2AR or anti-GAPDH.
